# Targeted Phosphoinositides Analysis Using High-Performance Ion Chromatography-Coupled Selected Reaction Monitoring Mass Spectrometry

**DOI:** 10.1021/acs.jproteome.1c00017

**Published:** 2021-05-03

**Authors:** Hilaire Yam Fung Cheung, Cristina Coman, Philipp Westhoff, Mailin Manke, Albert Sickmann, Oliver Borst, Meinrad Gawaz, Steve P. Watson, Johan W. M. Heemskerk, Robert Ahrends

**Affiliations:** † Leibniz-Institut für Analytische Wissenschaften-ISAS-e.V., 44227 Dortmund, Germany; ‡ Institute of Cardiovascular Sciences, Institute of Biomedical Research, College of Medical and Dental Sciences, University of Birmingham, Edgbaston, Birmingham B15 2TT, U.K.; § Department of Biochemistry, Cardiovascular Research Institute Maastricht (CARIM), Maastricht University, 6229 ER Maastricht, The Netherlands; ∥ Department of Analytical Chemistry, Faculty of Chemistry, University of Vienna, 1090 Wien, Austria; ⊥ Department of Cardiology and Cardiovascular Medicine, University of Tübingen, 72076 Tübingen, Germany

**Keywords:** phosphoinositides, targeted lipidomics, ion chromatography

## Abstract

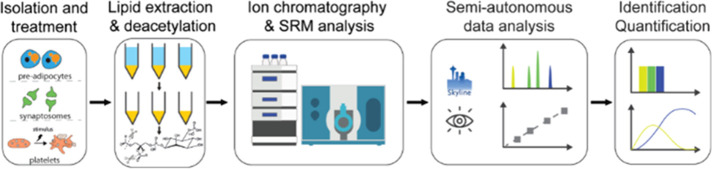

Phosphoinositides are minor components of cell membranes, but play crucial roles in numerous signal transduction pathways. To obtain quantitative measures of phosphoinositides, sensitive, accurate, and comprehensive methods are needed. Here, we present a quantitative targeted ion chromatography–mass spectrometry-based workflow that separates phosphoinositide isomers and increases the quantitative accuracy of measured phosphoinositides. Besides testing different analytical characteristics such as extraction and separation efficiency, the reproducibility of the developed workflow was also investigated. The workflow was verified in resting and stimulated human platelets, fat cells, and rat hippocampal brain tissue, where the LOD and LOQ for phosphoinositides were at 312.5 and 625 fmol, respectively. The robustness of the workflow is shown with different applications that confirms its suitability to analyze multiple less-abundant phosphoinositides.

## Introduction

Phosphoinositides are one of the highly diverse glycerophospholipid subcategories. Reversible phosphorylation of the myo-inositol headgroup of phosphatidylinositol gives rise to seven distinct phosphoinositides positional isomers in biological systems, namely, PtdIns3P, PtdIns4P, PtdIns5P, PtdIns(3,4)P_2_, PtdIns(3,5)P_2_, PtdIns(4,5)P_2_, and PtdIns(3,4,5)P_3_. Phosphoinositides are versatile signaling molecules crucial in signal transduction, especially PtdIns(4,5)P_2_ and PtdIns(3,4,5)P_3_, which play a central role in the InsP_3_/DAG pathway^1^ and the PI3K/Akt pathway.^2,3^ They also act as constitutive signals that help define organelle identity and regulate protein localization and membrane trafficking.^4^ Hence, each of the phosphoinositides has a specific spatial distribution pattern among organelles. For example, PtdIns4P is predominantly found at the Golgi complex, while PtdIns(3,4)P_2_, PtdIns(4,5)P_2_, and PtdIns(3,4,5)P_3_, which play important roles in signal transduction, are concentrated at the plasma membrane. PtdIns3P and PtdIns(3,5)P_2_, which regulate endosome fission and fusion, are concentrated in early endosomes and late endosomes, respectively.^5^

Consistent with the central roles of these lipids, mutations in the network of enzymes responsible for their synthesis and degradation have been linked to a variety of diseases.^6^ Phosphoinositide 3-kinase inhibitors have been proposed as novel antiplatelet agents, to prevent thrombotic events in stroke and cardiovascular diseases.^7^ Defects in synaptojanin-1, a PtdIns(4,5)P_2_ phosphatase that is widely expressed in neurons, have been linked to Alzheimer’s disease and Down’s syndrome,^8,9^ while mutations in SHIP2, a PtdIns(3,4,5)P_3_ phosphatase, have been associated with type 2 diabetes.^10^

This has spurred the development of methods capable of measuring changes in these lipids. Nevertheless, despite the importance of phosphoinositides, their identification and quantification remain a major challenge, largely due to their low abundance and high polarity.^2^ In the 1990s, their qualitative and quantitative analyses were conducted through phosphoinositide deacylation combined with the use of thin-layer chromatography for enrichment, anion-exchange chromatography for separation, and ^32^P radioactive labeling for detection.^11−13^ The method has several limitations such as use of radioactive materials, the challenge in labeling primary cells and tissues, and the number of steps involved.

Nonradioactive methods using tandem mass spectrometry (MS/MS) have been then developed to detect and quantify phosphoinositides. In 2002, Wenk et al. introduced the use of MS/MS for phosphoinositides analysis.^14^ The phosphoinositides were extracted by acidified chloroform/methanol, and piperidine was applied as an ion-pairing agent. Direct infusion and the precursor-ion scan mode targeting the inositol phosphate fragment ions were utilized for phosphoinositides analysis. The method could not however differentiate the phosphoinositides positional isomers, was unable to identify PtdInsP3, and had relatively low sensitivity for PtdInsP and PtdInsP2, at approximately 50 and 150 pmol, respectively.

Nowadays, phosphoinositides are most often measured by reversed-phase liquid chromatography coupled to tandem mass spectrometry (RPLC-MS/MS). In brief, phosphoinositides are extracted by acidified chloroform/methanol^15−19^ or acidic *n*-butanol/chloroform extraction.^20^ The extracted phosphoinositides are then derivatized with TMS-diazomethane to methylate the phosphate groups,^16,19,20^ deacylated with methylamine to remove the acyl chains and produce glycerophosphoinositol phosphates (GroPInsP),^18^ or just directly analyzed without any derivatization.^15,17^ In the case of deacylated or underivatized phosphoinositides, RPLC-electrospray ionization (RPLC-ESI) is the method of choice and has the advantage of separating phosphoinositides positional isomers.^15,18^ The addition of an ion-pairing reagent is also needed to shield the highly polar phosphate group and facilitate isomers separation using reversed-phase chromatography, which may contaminate the MS, cause ion suppression, and affect the ionization pattern of ions when it is used for other purposes.^21,22^ RPLC-ESI separation of methylated phosphoinositides is robust and sensitive, but it is unable to differentiate the phosphoinositides positional isomers.^16,20^ Differentiating the positional isomers is possible for methylated phosphoinositides if direct infusion is used, but at the cost of adding high concentrations of lithium ions, as well as the need of sophisticated analysis to determine the positional isomers ratios based on the ratio of lithiated ions.^19^

Recent developments in ion chromatography (IC) allow conductivity suppression by continuous online removal of high salt concentrations leaving the analytes in pure water, which permits online coupling of IC with MS. So far, IC was utilized with tandem mass spectrometry (IC-MS/MS) for untargeted metabolic profiling and targeted screening and quantification of metabolites such as carbohydrates, organic acids, sugar phosphates, and nucleotides in different biological matrices.^23−25^ However, its application to phosphoinositides analysis has not yet been fully explored.

In this study, we report the use of IC-MS/MS to resolve the deacylated phosphoinositides positional isomers for absolute quantification of these isomers with high sensitivity in tissues and cells. We believe that the developed method will greatly facilitate the analysis of phosphoinositides and bring us an important step closer to the global understanding of phosphoinositides signaling.

## Experimental Section

### Materials

Chemicals and reagents were obtained from the following sources: MS-grade methanol (MeOH) from Biosolve (Valkenswaard, The Netherlands); formic acid, 37% hydrochloric acid (HCl), chloroform (CHCl_3_), and methylamine in MeOH from Sigma-Aldrich (Steinheim, Germany); sodium chloride (NaCl), 1-butanol, and isopropanol (IPA) from Merck (Darmstadt, Germany); Tris(hydroxymethyl)-aminomethane (Tris) from Applichem (Darmstadt, Germany); sodium dodecyl sulfate (SDS) from Roth (Karlsruhe, Germany); 16:0/16:0 PtdIns4P and 16:0/16:0 PtdIns(4,5)P2 α-fluorovinylphosphonate (PtdIns(4,5)P_2_-FP) from Echelon Biosciences (Salt Lake City, UT); and 17:0/20:4 PtdIns3P, 18:1/18:1 PtdIns(3,4)P_2_, 18:1/18:1 PtdIns(4,5)P_2_, 18:1/18:1 PtdIns(3,5)P_2_, and 17:0/20:4 PtdIns(3,4,5)P_3_ from Avanti Polar Lipids (Alabaster, AL). Ultrapure water (18 MΩ cm at 25 °C) was obtained from an Elga Labwater system (Lane End, U.K.). Bicinchoninic acid (BCA) assay was purchased from Thermo Scientific (Schwerte, Germany). Platelets were activated using collagen-related peptide (CRP, Richard Farndale, University of Cambridge, U.K.) or thrombin from human plasma (Roche, Germany).

### Ethical Regulations for Animal Samples

Four-week-old male C57BL/6J mice (Charles River, Germany) were used. Male Wistar rats were used at the age of 10 weeks in six independent preparations. The animals were euthanized, and the hippocampi were dissected. All animal experimentations were performed in accordance with the ARRIVE guidelines for animal experimentation^26^ and EU regulations, and approved by the local ethical committee.

### Ethical Regulations for Human Samples

All volunteers gave informed consent for blood samples. The platelet study was approved by the institutional ethics committee (270/2011BO1) at the University Hospital Tübingen (Germany) and complied with the Declaration of Helsinki and good clinical practice guidelines.

### Preparation of Human Platelets

Blood from four individual healthy volunteers was collected to obtain four individual samples in ACD buffer (70 mM citric acid, 116 mM sodium citrate, 111 mM glucose, pH 4.6) and centrifuged at 200*g* for 20 min. The obtained platelet-rich plasma was added to modified Tyrode-HEPES (*N*-2-hydroxyethyl-piperazone-*N*′2-ethanesulfonic acid) buffer (137 mM NaCl, 2 mM KCl, 12 mM NaHCO_3_, 5 mM glucose, 0.3 mM Na_2_HPO_4_, 10 mM HEPES, pH 6.5). After centrifugation at 900*g* for 10 min and removal of the supernatant, the resulting platelet pellet was resuspended in Tyrode-HEPES buffer (pH 7.4, supplemented with 1 mM CaCl_2_).

### Platelet Stimulation Experiment

Freshly isolated and resuspended human platelets in 100 μL at a concentration of 1 × 10^6^ platelets/μL were stimulated with either 0.01 U/mL thrombin or 1 μg/mL CRP for 5 min. After centrifugation for 5 min at 640*g* at 25 °C, the pellets were shock-frozen in liquid nitrogen and stored at −80 °C.

### Cell Culture

Mesenchymal stem cells (OP9) were grown following a previously published protocol.^27^ Briefly, the cells were grown in MEM with l-glutamine, 20% FBS, and 100 U/mL penicillin/streptomycin. Cultures were maintained at 37 °C in humidified atmosphere with 5% CO_2_, and the medium was renewed every 4 days. After reaching 80% confluence, the cells were trypsinized, washed with PBS, and collected from culture dish. The cells were aliquoted to 1 × 10^7^ cells per sample, centrifuged at 400*g* for 5 min, the supernatant was removed, and the cell pellet were snap-frozen in liquid nitrogen.

### Membrane Preparation from Rat Hippocampal Brain Tissue

Subcellular fractionation of rat hippocampus was performed as described earlier.^28^ Rat hippocampal tissue (3.5 g) was homogenized in 10 mL/g buffer A (0.32 M sucrose, 5 mM HEPES, pH 7.4) including protease inhibitor cocktail (PI) and phosphatase inhibitor (PhosSTOP) and centrifuged at 1000*g* for 10 min. The pellet was rehomogenized and centrifuged in buffer A. The resulting pellet 1 containing nuclei and cell debris was discarded, and the supernatants were combined. The combined supernatants were centrifuged at 12 000*g* for 20 min (Sorvall RC6, F13-14 x 50cy rotor). The pellet P2 was rehomogenized in buffer A and centrifuged as previously at 12 000*g* for 20 min. The resulting pellet was collected as the hippocampus heavy membrane fraction.

### Lipid Extraction

Acidified chloroform/methanol (CHCl_3_/MeOH) extraction was carried out following the protocol of Clark et al.^16^ For platelet samples, after the addition of 242 μL of CHCl_3_, 484 μL of MeOH, 23.6 μL of 1 M HCl, 170 μL water, and internal standard (100 pmol of PtdIns(4,5)P_2_-FP) to the cell pellets containing 1 × 10^8^ platelets, the mixture was allowed to stand at room temperature for 5 min with occasional vortexing. Next, 725 μL of CHCl_3_ and 170 μL of 2 M HCl were added to induce phase separation and the samples were centrifuged at 1500*g* for 5 min at room temperature (Eppendorf, Hamburg, Germany). This created a two-phase system with an upper aqueous layer and a protein interface. Then, the lower organic layer was transferred to another tube and dried under a continuous stream of nitrogen (1 L/min N_2_ at 25 °C).

For pre-adipocytes and rat hippocampal heavy membrane fraction, after the addition of 242 μL of CHCl_3_, 484 μL of MeOH, 25 μL of 50 mM NaOH, 170 μL of water, and the internal standard (2 nmol of PI(4,5)P_2_-FP) to the cell pellets, the mixture was vortexed and sonicated until homogenization. Afterward, 725 μL of CHCl_3_ was added and the samples were centrifuged at 1500*g* for 5 min at room temperature. The resulting lower phase containing neutral lipids was removed without disturbing the upper aqueous phase and protein interphase. Next, 170 μL of 2 M HCl, 333 μL of MeOH, and 667 μL of CHCl_3_ were added to the remaining phase and the mixture was allowed to stand at room temperature for 5 min with occasional vortexing. The samples were then centrifuged at 1500*g* for 5 min at room temperature. Next, the lower organic layer was transferred to another tube and dried under a continuous stream of nitrogen (1 L/min N_2_ at 25 °C).

The lipid extracts were then deacylated following the protocol of Jeschke et al.^18^ The dried lipid extracts were resuspended in 50 μL of methylamine in methanol/water/1-butanol (46:43:11) and incubated at 53 °C for 50 min in a thermomixer at 1000 rpm (Thermomixer Comfort; Eppendorf, Hamburg, Germany). Then, 25 μL of cold IPA was added to the mixture and the mixture was dried under a continuous stream of nitrogen to obtain dried lipid extracts (1 L/min N_2_ at 25 °C). The dried and deacylated lipid extract was resuspended in 50 μL of water and stored at −80 °C prior to further analysis.

### Protein Concentration Determination

Methanol (1200 μL) was added to the remaining protein interphase and aqueous upper phase, and the mixture was incubated at −80 °C for 3 h. Then, the mixture was centrifuged at 19 000*g* for 30 min at 4 °C, the supernatant was removed, and the remaining protein pellet was dried under the fume hood. The resulting protein pellet was then resuspended in 1% SDS, 150 mM NaCl, 50 mM Tris (pH 7.8), and the protein concentration was determined using the BCA assay.

### IC-MS/MS

IC-MS/MS was conducted using a Dionex ICS-5000 instrument (Thermo Fischer Scientific, Darmstadt, Germany) connected to a QTRAP 6500 instrument (AB Sciex, Darmstadt, Germany) that was equipped with an electrospray ion source (Turbo V ion source). Chromatographic separation was accomplished with a Dionex IonPac AS11-HC column (250 mm × 2 mm, 4 μm; Thermo Fischer Scientific) fitted with a guard column (50 mm × 2 mm, 4 μm; Thermo Fisher Scientific). A segmented linear gradient was used for separation of GroPInsP: Initial 15 mM potassium hydroxide (KOH), held at 15 mM KOH from 0.0 to 5.0 min, 15–25 mM KOH from 5.0 to 15.0 min, 50–65 mM KOH from 15.0 to 30.0 min, 100 mM KOH from 30.0 to 34.0 min, 10 mM KOH from 34.0 to 38.0 min, 100 mM KOH from 38.0 to 42.0 min, and 15 mM KOH from 42.0 to 45.0 min. The IC flow rate was 0.38 mL/min, supplemented post-column with 0.15 mL/min makeup flow of 0.01% FA in MeOH. The temperatures of the autosampler, column oven, and ion suppressor were set at 10, 30, and 20 °C, respectively. The injector needle was automatically washed with water, and 5 μL of each sample was loaded onto the column.

The following ESI source settings were used: curtain gas, 20 arbitrary units; temperature, 400 °C; ion source gas I, 60 arbitrary units; ion source gas II, 40 arbitrary units; collision gas, medium; ion spray voltage, −4500 V; declustering potential, −150 V; entrance potential, −10 V; and exit potential, −10 V. For scheduled selected reaction monitoring (SRM), Q1 and Q3 were set to unit resolution. The collision energy was optimized for each GroPInsP by direct infusion of the corresponding deacylated standard. The scheduled SRM detection window was set to 3 min, and the cycle time was set to 1.5 s. Data were acquired with Analyst version 1.6.2 (AB Sciex). Skyline (64-bit, 3.5.0.9319) was used to visualize results, integrate signals over time, and quantify all lipids that were detected by MS.^29^

## Results and Discussion

### Establishing Profiling Strategies for Phosphoinositides

**Figure 1:**
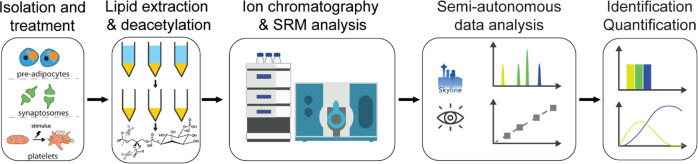
Schematic diagram of targeted phosphoinositides analysis workflow using an ion chromatography-QTRAP system. Phosphoinositides in platelets, OP9 cells, and rat hippocampal brain tissue were extracted by acidified chloroform/methanol and then deacylated with methylamine. The phosphoinositides were then separated on an anion-exchange column by a KOH gradient and analyzed using the SRM approach. Data analysis was conducted using Skyline, and the absolute quantities of each positional isomers were determined.

We present here an improved quantitative IC-MS/MS workflow for phosphoinositides analysis, which includes the addition of standards, a modified extraction and deacylation procedure, and an optimized IC method, resulting in a comprehensive quantitative workflow (Figure 1).

#### Extraction Strategies for Phosphoinositides

We reviewed and compared earlier extraction strategies of phosphoinositides. As a base, we chose the acidified chloroform/methanol strategy described in Clark et al.,^16^ which protonates the phosphate groups on phosphoinositides headgroups to increase their solubility in the organic phase. We then deacylated the phosphoinositides with methylamine to remove the fatty acid chain and produce GroPInsP, dried the GroPInsP with nitrogen stream, and reconstituted the extract in water prior to IC-MS/MS analysis, as modified from the protocol detailed by Jeschke et al.^18^

**Figure 2:**
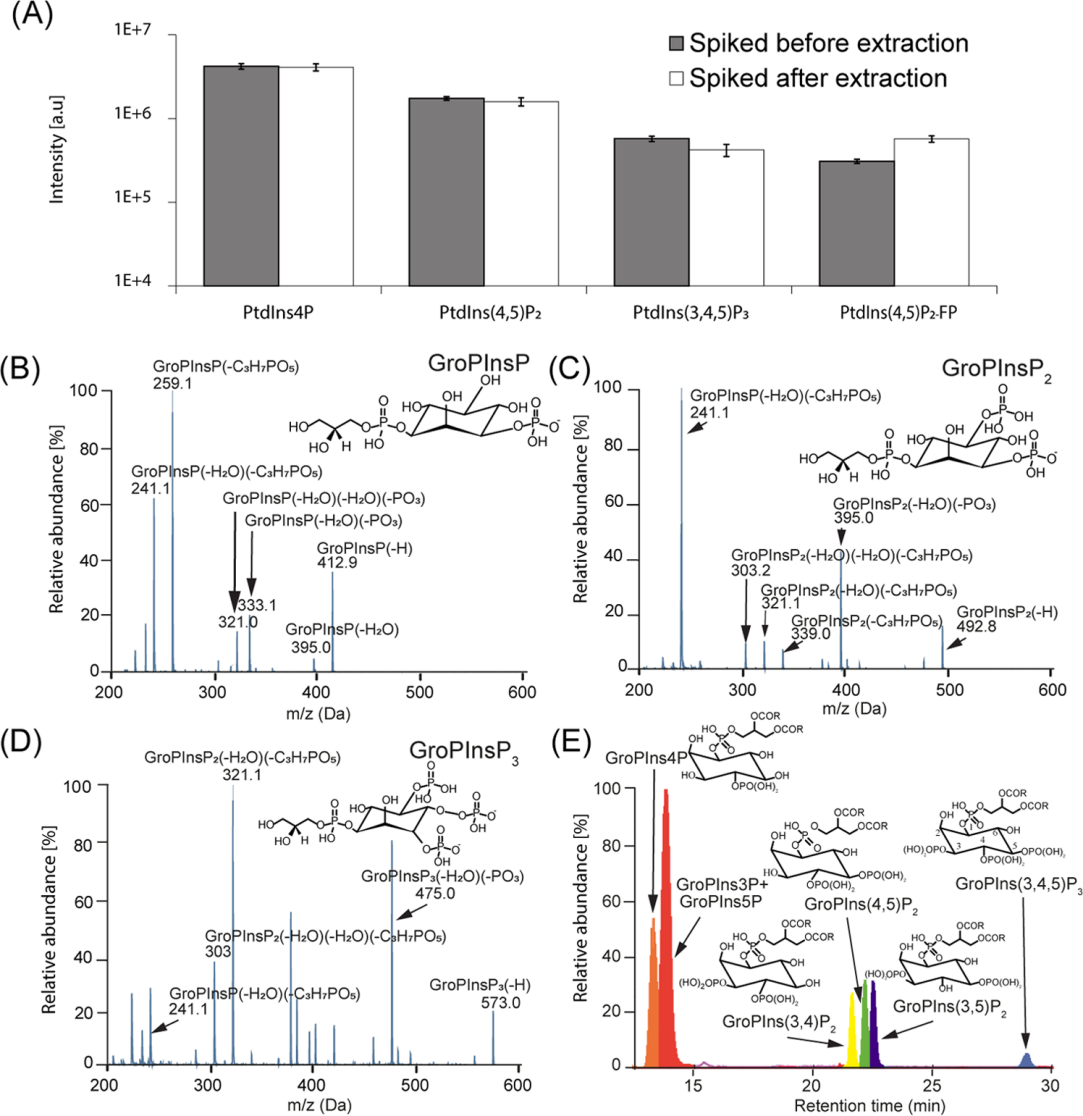
Extraction efficiency, elution profile, and MS/MS spectra of phosphoinositide/GroPInsP species. (A) Bar chart showing the peak area of the phosphoinositides with endogenous and synthetic standards spiked into unstimulated human platelets before (black) or after (white) extraction in triplicate. The recoveries for PtdIns4P, PtdIns(4,5)P_2_, PtdIns(3,4,5)P_3_, and synthetic standard PtdIns(4,5)P_2_-FP were 103 ± 12, 109 ± 11, 134 ± 13, and 54.5 ± 20%, respectively (*n* = 3 technical replicates). (B–D) Fragment-ion spectra of (B) GroPInsP, (C) GroPInsP_2_, and (D) GroPInsP_3_ obtained on QTRAP. Collision-induced dissociation of these headgroups led to the formation of fragments that lost −C_3_H_7_O_5_P, −H_2_O, or −O_3_P molecules. (E) SRM extracted-ion chromatogram (XIC) of deacylated phosphoinositide standard mixture. The three GroPInsP_2_ positional isomers were separated using the optimized gradient. GroPIns3P and GroPIns5P could not be resolved, but GroPIns4P could be separated. The structure of each GroPInsP is also shown.

The extraction efficiency of the strategy was then validated by spiking synthetic internal standards PtdIns(4,5)P_2_-FP into unstimulated human platelets before or after extraction (Figure 2A). PtdIns(4,5)P_2_-FP is a metabolically stabilized analogue of PtdIns(4,5)P_2_, which contains a fluorovinylphosphonate group instead of a phosphodiester bond (Figure S4). The recoveries for PtdIns4P, PtdIns(4,5)P_2_, PtdIns(3,4,5)P_3_, and synthetic standard PtdIns(4,5)P_2_-FP were determined to be 103 ± 12, 109 ± 11, 134 ± 13, and 54.5 ± 20%, respectively, suggesting that the phosphoinositides were analyzed with good recoveries.

#### Optimization of IC Method for Phosphoinositides Analysis

The extracted and deacylated phosphoinositides headgroups were separated by IC, which was equipped with an anion-exchange column, and eluted with a KOH gradient according to the negative charges on the analyte molecules. After the removal of highly concentrated hydroxide ions by the ion suppressor, the output flow was mixed with a makeup flow of 0.01% formic acid in MeOH and analyzed in an enhanced product-ion scan experiment monitoring the fragment-ion spectra of the selected precursor ions, as shown in Figure 2B−D. The use of IC instead of RPLC eliminated the need of adding ion-pairing reagents, which could contaminate the MS Instruments, while providing good separation to the GroPInsP isomers (Figure 2E).

**Figure 3:**
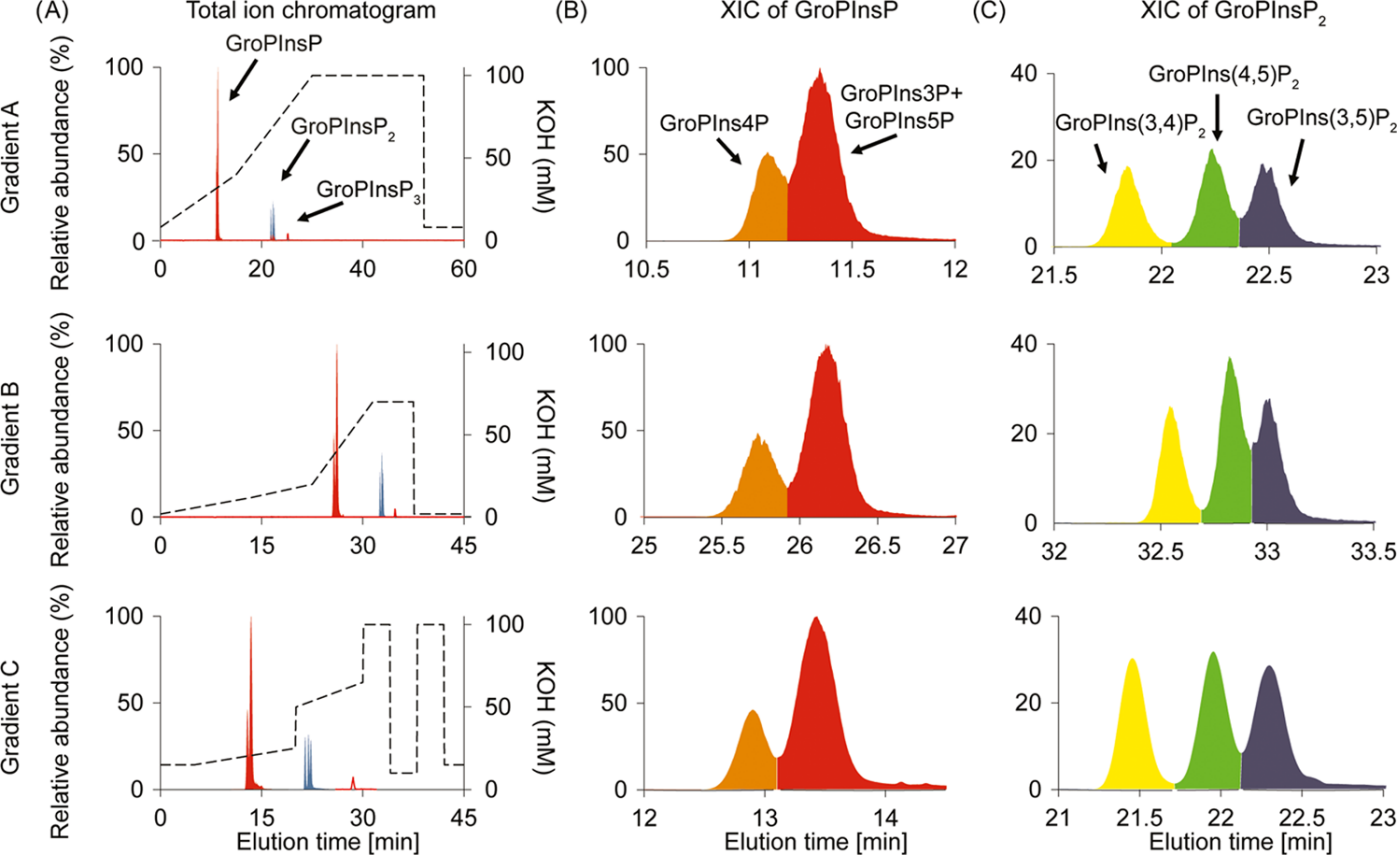
Comparison of three LC gradients. (A) Total ion chromatogram (TIC) of the GroPInsP and the IC gradient of gradients A, B, and C. The left axis shows the relative abundance of the ions in the TIC, and the right axis shows the concentration of KOH used in each gradient. (B) XIC of the GroPInsP. For GroPIns4P and GroPIns3P, gradients C (resolution (R), 100%) and B (R, 100%) yielded separation that was better than that of gradient A (R, 70.2%). All three gradients failed to resolve GroPIns3P from GroPIns5P. (C) XIC of GroPInsP_2_. For GroPIns(3,4)P_2_, GroPIns(4,5)P_2_, GroPIns(4,5)P_2_, and GroPIns(3,5)P_2_, gradients C (R, 100 and 100%) and A (R, 104 and 96%) yielded separation that were better than that of gradient B (R, 75 and 70%). The IC gradients of gradients A, B, and C are derived from refs (23) and (25) and this study, respectively.

We optimized the IC gradient for phosphoinositide separation to achieve a comprehensive analysis of individual GroPInsP isomers. Based on the results from previous studies, we evaluated different ion chromatography separation gradients that are currently used in the field for metabolomics analysis, and the results from two of these are reported (Figure 3).^23,25^ Gradients B [resolution (R), 100%] and C (R, 100%) yielded separation that was better than that of gradient A (R, 70.2%) for GroPIns4P and GroPIns3P. For GroPIns(3,4)P_2_, GroPIns(4,5)P_2_, and GroPIns(4,5)P_2_, GroPIns(3,5)P_2_, Gradients C (R, 100 and 100%) and A (R, 104 and 96%) yielded separation that was better than that of gradient B (R, 75 and 70%). All tested gradients were unable to resolve GroPIns3P from GroPIns5P, which is rooted in their structural similarity. The results of our HPLC gradient evaluations indicate that the use of the optimized segmented linear gradient C is the best choice for the separation of both GroPInsP and GroPInsP_2_ classes.

**Figure 4:**
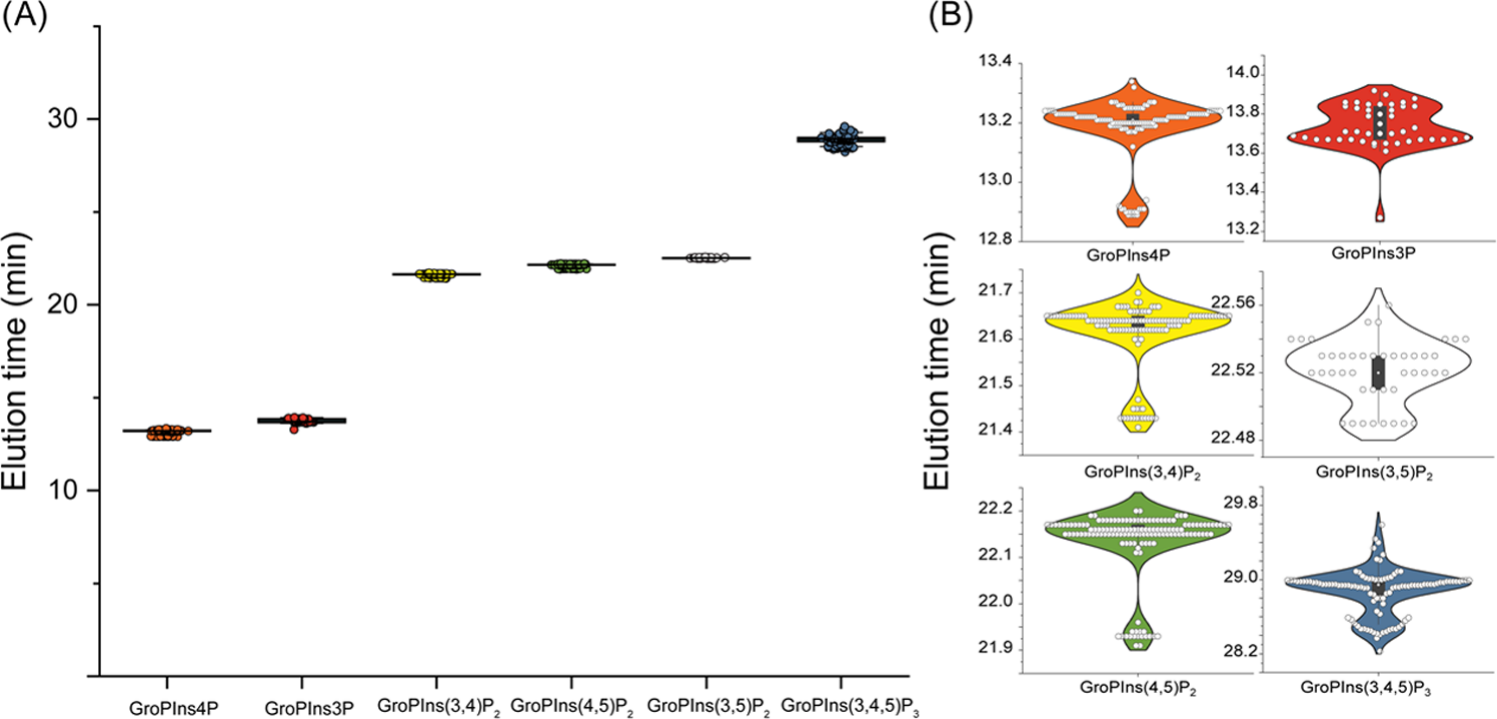
Elution time reproducibility of the developed workflow. (A) Dot plot and (B) violin plot showing the elution time of the GroPInsP in a large number of injections of standards and phosphoinositide-containing samples derived from human platelets, OP9 cells, and rat hippocampal brain tissue (*n* = 43–141 LC-MS runs).

To prove the robustness of the developed method, the stability of the IC gradient was validated across 120 injections of standards and phosphoinositides containing samples derived from human platelets, OP9 cells, and rat brain fractions (Figure 4). The retention times of GroPIns4P, GroPIns(4,5)P_2_, GroPIns(3,4,5)P_3_, and GroPIns(4,5)P_2_-FP averaged at 13.18 ± 0.11, 22.13 ± 0.08, 28.87 ± 0.24, and 22.03 ± 0.09 min, respectively, suggesting that the IC method for GroPInsP separation is highly reproducible.

#### Quantification of Phosphoinositides Using Targeted Mass Spectrometry

Targeted analysis of phosphoinositides was performed using an SRM approach. Molecular ions selected in the first quadrupole were fragmented in the second quadrupole using collision parameters optimized to give the highest fragment ions intensity. The collision energy was chosen to be −32 eV for GroPIns(3,4,5)P_3_ and −27 eV for all other precursor molecules (Table S1). The deacylation of the phosphoinositides removed the fatty acyl chain, making it more hydrophilic and thus facilitating its separation through IC, simplifying the mass spectra and allowing the isomer analysis at the class level. However, some of the phosphoinositides headgroups have the same number of phosphate groups and similar MS^2^ fragmentation patterns that were indistinguishable (Figure S1). Therefore, an optimized LC gradient was necessary to separate and differentiate the different GroPInsP and GroPInsP_2_ isomers (Figure 2E, gradient C in Figure 3A–C).

**Figure 5:**
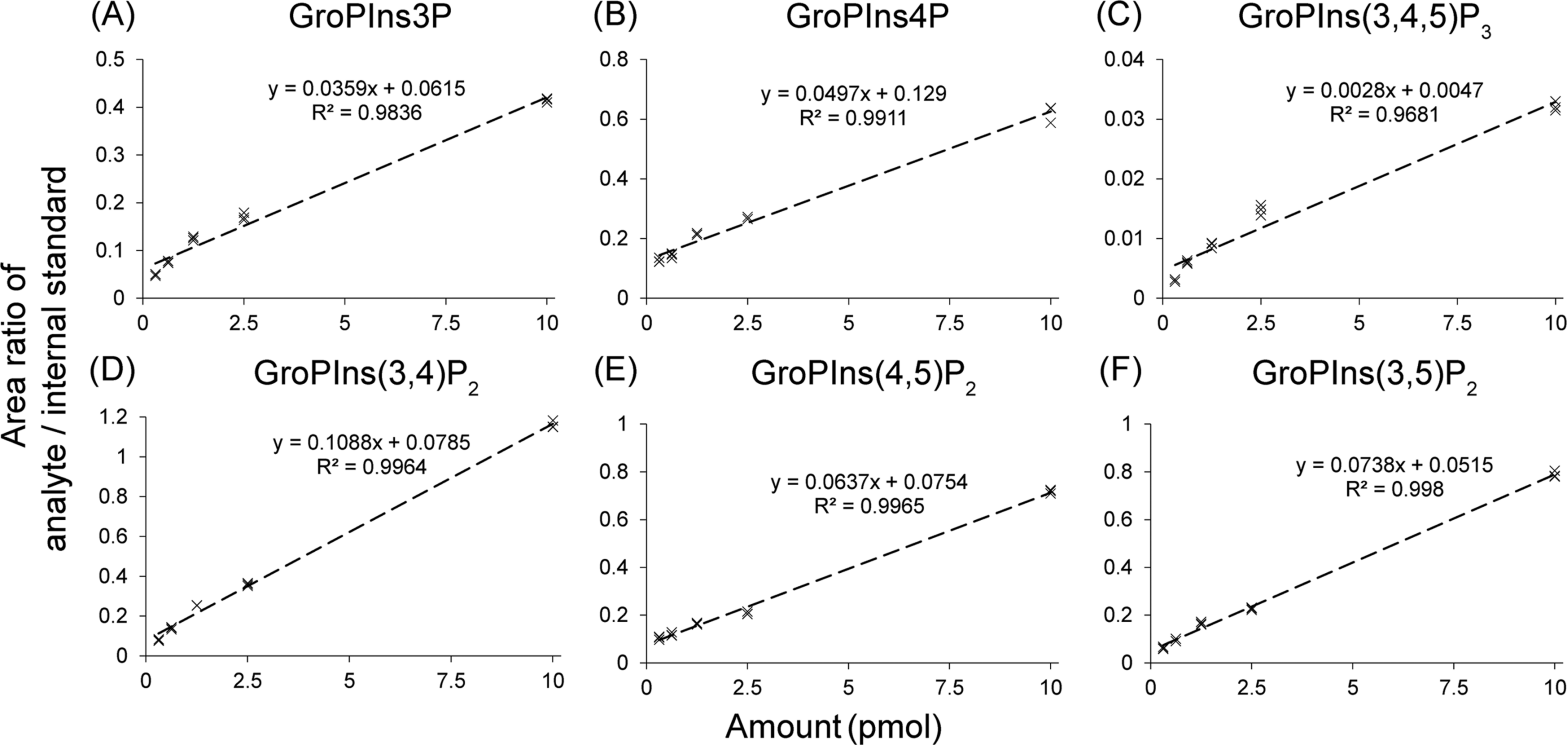
Internal standard calibration curve for absolute quantification of GroPInsP. A known amount of phosphoinositides and 100 pmol of internal standard were spiked into unstimulated human platelets extracts, and then the phosphoinositides were extracted and deacylated using the workflow. The areas under the extracted-ion chromatogram peak of (A) GroPIns3P, (B) GroPIns4P, (C) GroPIns(3,4,5)P_3_, (D) GroPIns(3,4)P_2_, (E) GroPIns(4,5)P_2_, and (F) GroPIns(3,5)P_2_ were divided by that of the internal standard, and the area ratio was plotted against the amount of the species spiked. The resulting calibration curve, data points, and *R*^2^ value are shown (*n* = 3 technical replicates).

To quantify the phosphoinositides in complex biological matrices, we used the internal calibration curve approach to achieve maximum accuracy. We spiked known amounts of phosphoinositides and 100 pmol of internal standard PtdIns(4,5)P_2_-FP into 1 × 10^8^ unstimulated human platelets. The area ratio was calculated by dividing the highest-intensity fragment at −27 eV for GroPInsP and GroPInsP_2_ and at −32 eV for GroPInsP_3_ by that of the internal standard at −27 eV. The resulting calibration curves ranged from 312.5 fmol to 10 pmol with a high degree of linearity (*R*^2^ ≈ 0.99) (Figure 5). These results indicate that the use of a synthetic internal standard, PtdIns(4,5)P_2_-FP, to correct for recovery through the extraction, deacylation, and the IC-SRM assay is a very sensitive and robust method to absolutely quantify all phosphoinositides species in cells.

The method’s limit of detection (LOD) and limit of quantification (LOQ) were considered as the analyte concentration required to produce a signal intensity that is 3 times or 10 times higher than the noise signal. Using these criteria, GroPIns(3,4,5)P_3_ could be detected in unstimulated extracts with as little as 312.5 fmol (S/N > 3) and quantified at 625 fmol (S/N > 10) (Figure S2).

Compared with the most sensitive method reported so far, which has a limit of detection of 250 pg (equivalent to 250 fmol) C18:0/C20:4-PtdIns(3,4,5)P_3_,^16^ the current method provided comparable sensitivity to positional isomers resolution.

### Phosphoinositides Profile in Complex Biological Samples

**Figure 6:**
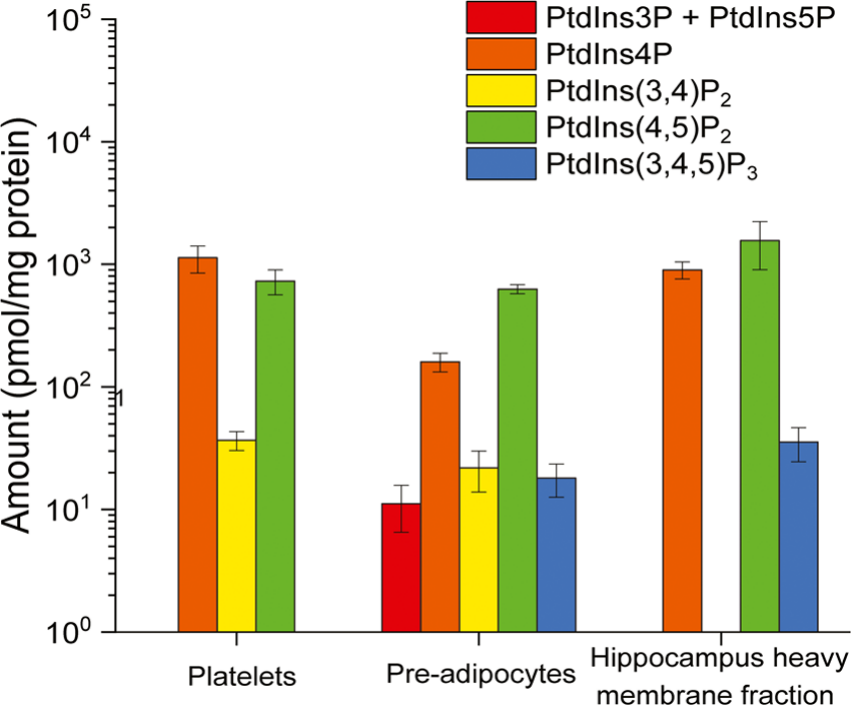
Resting phosphoinositides amount in platelets, OP9 pre-adipocytes, and rat hippocampus heavy membrane fraction. Bar chart showing phosphoinositides profile in different biological samples, including human platelets, OP9 pre-adipocytes cell culture, and rat hippocampus heavy membrane fraction (*n* = 3 biological replicates).

**Figure 7:**
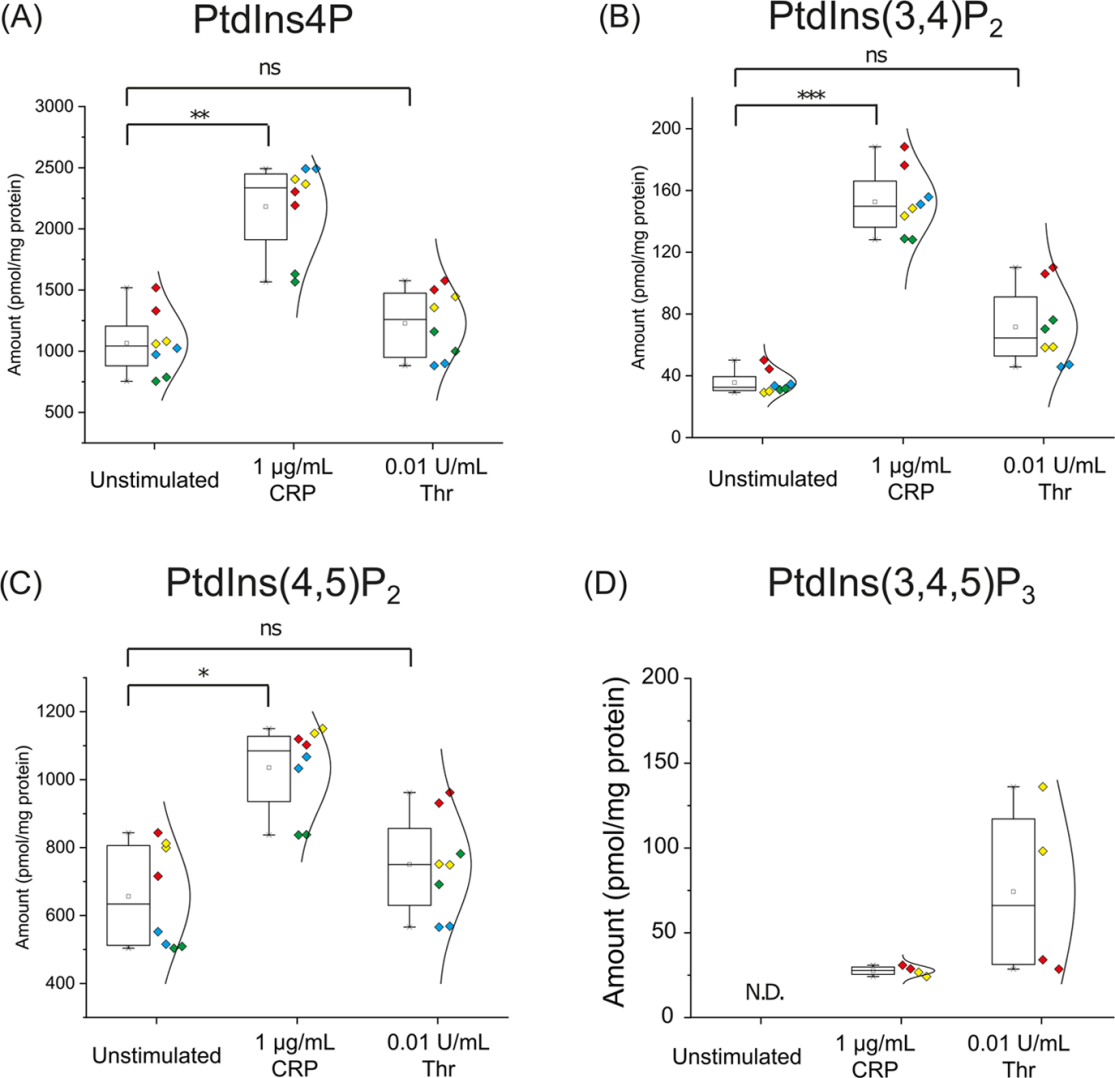
Effect of CRP and thrombin on human platelets phosphoinositides profile. Box plot showing changes in the phosphoinositides species, including (A) PtdIns4P, (B) PtdIns(3,4)P_2_, (C) PtdIns(4,5)P_2_, and (D) PtdIns(3,4,5)P_3_ in human platelets after thrombin and CRP stimulation, quantified by internal calibration curve, with a half-violin plot showing the distribution of phosphoinositides of each donor. Each color corresponds to four separate donors as biological replicates, and each dot corresponds to one of the technical duplicates. *P < 0.05, **P < 0.01, and ***P < 0.001 indicate statistically significant differences. ns, not significant. N.D., not detectable (*n* = 4 biological replicates).

To illustrate the effectiveness of our workflow, we applied it to complex biological samples including cell culture (pre-adipocyte OP9 cells), rat brain tissue, and human platelets; successfully quantified the phosphoinositides positional isomers (Figures 6 and S3); and quantified rapid phosphoinositides profile changes in platelets upon ligand stimulation (Figure 7).

#### Phosphoinositides Profile in Resting and Stimulated Human Platelets

In resting platelets, three phosphoinositides species, PtdIns4P, PtdIns(3,4)P_2_, and PtdIns(4,5)P_2_, were identified and quantified, at 94.7 ± 11.1 pmol/1 × 10^8^ platelets, 3.1 ± 0.2 pmol/1 × 10^8^ platelets, and 59.2 ± 12.4 pmol/1 × 10^8^ platelets, respectively (Figure S3A). Previous studies, which used a combination of radioactive labeling, TLC, and HPLC to analyze phosphoinositides in platelets, determined the level of PtdIns4P and PtdIns(4,5)P_2_ to be 150–245 pmol/1 × 10^8^ platelets and 55–139 pmol/1 × 10^8^ platelets, respectively. The phosphoinositides level determined in the present study showed high consistency with these reports, thus further illustrating the reliability of the introduced workflow (Figure S3).^30−32^

To prove that the workflow can also catch the transient changes in phosphoinositides signaling, we analyzed the phosphoinositides profile in stimulated human platelets and assessed the effect of different agonists such as collagen-related peptide (CRP) and thrombin on phosphoinositides metabolism (Figure 7). CRP and thrombin treatment of platelets has been previously reported to activate platelets through platelet receptor glycoprotein VI (GPVI) and protease-activated receptors (PARs), respectively.^33,34^ It has been reported that 10 min CRP treatment in human platelets increased the level of PtdIns4P by 1.5-fold and PtdIns(4,5)P_2_ by 1.25-fold, as well as increased the level of PtdIns(3,4,5)P_3_.^35^ However, that study was unable to differentiate PtdIns(3,4)P_2_ from PtdIns(4,5)P_2_, resulting in loss of important signaling information. Knowing and differentiating the levels of PtdIns(4,5)P_2_ and PtdIns(3,4)P_2_ would provide information about the formation and flux change of PtdIns(3,4,5)P_3_ because PtdIns(3,4)P_2_ is a product of SHIP1 and SHIP2, two PtdIns(3,4,5)P_3_ 5-phosphatases that are known to regulate the level of PtdIns(3,4,5)P_3_ in platelets.^36^

Here, we were able to absolutely quantify each individual species in unstimulated, CRP, and thrombin-stimulated platelets (Figure 7). Similar to previous studies, we found that the levels of PtdIns4P and PtdIns(4,5)P_2_ were increased by 2-fold, from 122.3 ± 27.8 to 243.7 ± 46.1 pmol/mg protein and 1.5-fold, from 78.8 ± 13.7 to 118.1 ± 20.5 pmol/mg protein, respectively, after 5 min of CRP treatment, which is due to the increased production via PtdIns4P toward PtdIns(4,5)P_2_ by phosphoinositides kinase PIP4K and PIP5K as previously reported.^30,37^ The most prominent change was observed for PtdIns(3,4)P_2_, which increased 4.5-fold, from 4.0 ± 0.8 to 17.1 ± 3.5 pmol/mg protein after 5 min of stimulation, resulting most probably from the dephosphorylation of PtdIns(3,4,5)P_3_ by phosphoinositides phosphatase SHIP or other poly-phosphatases present in platelets.^36,38^ On the other hand, the use of 0.01 U/mL thrombin as stimulus was unable to cause a significant change in PtdIns4P and PtdIns(4,5)P_2_ profiles, but it led to a 2-fold increase in PtdIns(3,4)P_2_, from 4.0 ± 0.8 to 8.2 ± 2.6 pmol/mg protein.

#### Phosphoinositides Profile in OP9 Pre-Adipocytes

OP9 cells are pre-adipocytes that can rapidly differentiate into different types of adipocytes.^39^ All phosphoinositides species could be identified, including PtdIns4P and PtdIns(4,5)P_2_ (Figure 6). The amounts of PtdIns3P + PtdIns5P, PtdIns4P, PtdIns(3,4)P_2_, PtdIns(4,5)P_2_, and PtdIns(3,4,5)P_3_ are 11.1 ± 4.6, 160 ± 4, 21.8 ± 8.0, 630 ± 53, and 18.0 ± 5.4 pmol/mg protein, respectively. The higher variety and the detection of PtdIns(3,4,5)P_3_ can be explained by the existence of insulin-like growth factor 1 (IGF1) in FBS in culture media, which stimulated the IGF1 receptor on pre-adipocyte surface and led to production of PtdIns(3,4,5)P_3_ and derived species.^40,41^ Previous studies have also reported the production of PtdIns(3,4,5)P_3_ in other pre-adipocyte cell lines such as 3T3-L1 upon IGF1 stimulation, which induced pre-adipocytes growth and survival.^42,43^ PtdIns3P, PtdIns5P, and PtdIns(3,4)P_2_ were also detected in this study, as the dephosphorylation product of PtdIns(3,4,5)P_3_.^36^

#### Phosphoinositides Profile in Rat Hippocampus

In the heavy membrane fraction enriched from rat hippocampus, we identified and quantified three phosphoinositides species, PtdIns4P, PtdIns(4,5)P_2_, and PtdIns(3,4,5)P_3_, at 901 ± 116, 1560 ± 540, and 6.8 ± 2.0 pmol/mg protein, respectively (Figure 6). Compared to previous studies that determined the levels of PtdIns4P and PtdIns(4,5)P_2_ to be 1400 and 3860 pmol/mg protein, respectively (assuming the protein content in rat hippocampus to be 114 mg/g protein^44^), the phosphoinositides levels determined in the present study showed high consistency with these reports, and is further able to detect PtdIns(3,4,5)P_3_ (Figure S3), demonstrating the higher sensitivity of the chosen approach.^45^

## Conclusions

In this study, we developed an IC-SRM-based workflow that significantly increases the isomer resolution in phosphoinositides analysis and applied it to study the phosphoinositide composition in platelets, pre-adipocytes, and rat hippocampus membrane fraction. Our workflow was able to separate the biologically relevant GroPInsPs isomers except GroPIns3P and GroPIns5P and achieved LOD and LOQ for phosphoinositides at 312.5 and 625 fmol, respectively, thereby providing absolute amounts of different isomers. The workflow improves sample preparation and analysis and thus yields a higher level of confidence for phosphoinositide separation and quantification. Application of the workflow for different cell types and tissues demonstrates its versatility and potential to unravel specific roles played by each of the phosphoinositides.

## Supporting Information Available

Fragment-ion spectra of GroPInsP_2_ (Figure S1); limit of detection and limit of quantification of PtdIns(3,4,5)P_3_ (Figure S2); comparison of the amount of the identified phosphoinositides species with the literature (Figure S3); structure of internal standard 16:0/16:0 PtdIns(4,5)P_2_ α-fluorovinylphosphonate (PtdIns(4,5)P_2_-FP) (Figure S4); and SRM transition list for the current study (Table S1) (PDF)
