# Supplementary material for: Targeted Phosphoinositides Analysis Using High-Performance Ion Chromatography-Coupled Selected Reaction Monitoring Mass Spectrometry
Source: J Proteome Res. 2021 May 3;20(6):3114–23. doi: 10.1021/acs.jproteome.1c00017 (PMC8280744; doi:10.1021/acs.jproteome.1c00017)
Supplement: Supplementary file 1 — pr1c00017_si_001.pdf [file pr1c00017_si_001.pdf]

Supporting Information

Targeted Phosphoinositides Analysis Using High Performance Ion Chromatography-Coupled Selected Reaction Monitoring Mass Spectrometry

Hilaire Yam Fung Cheung[1,2,3], Cristina Coman[1,4], Philipp Westhoff[1], Mailin Manke[5], Albert Sickmann[1],Oliver Borst[5], Meinrad Gawaz[5], Steve P. Watson[2], Johan W.M. Heemskerk[3] and Robert Ahrends*[1,4]

[1]Leibniz-Institut für Analytische Wissenschaften-ISAS-e.V., 44227 Dortmund, Germany

[2]Institute of Cardiovascular Sciences, Institute of Biomedical Research, College of Medical and Dental Sciences, University of Birmingham, Edgbaston, Birmingham, B15 2TT, UK

[3]Department of Biochemistry, Cardiovascular Research Institute Maastricht (CARIM), Maastricht University, 6229 ER Maastricht, The Netherlands

[4]Department of Analytical Chemistry, Faculty of Chemistry, University of Vienna, 1090 Wien, Austria

[5]Department of Cardiology and Cardiovascular Medicine, University of Tübingen, 72076 Tübingen, Germany.

Table of contents:

S-2 Figure S1. Fragment ion spectra of $GroPInsP_2$.

S-3 Figure S2. Limit of detection and limit of quantification of PtdIns(3,4,5)P_3.

S-4 Figure S3. Comparison of amount of the identified phosphoinositides species with literature.

S-5 Figure S4. Structure of internal standard 16:0/16:0 PtdIns(4,5)P2 α-fluorovinyl-phosphonate (PtdIns(4,5)P2-FP).

S-6 Table S1. SRM transition list for the current study.

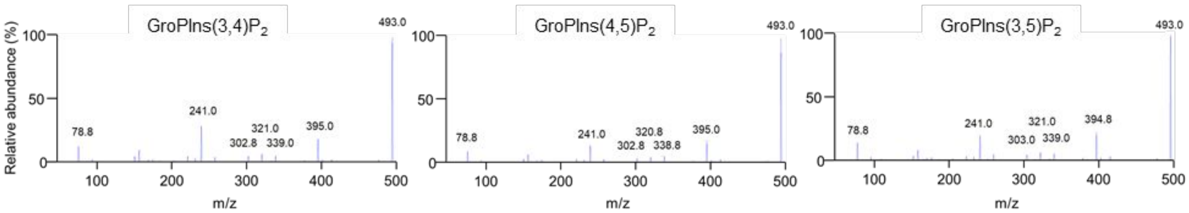

Figure S1. Fragment ion spectra of GroPInsP$_2$. Phosphoinositides standard were extracted, deacylated into glycerolphosphoinositol phosphates and injected into IC-MS/MS for analysis. The three GroPInsP$_2$ isomers had similar MS^2 fragmentation patterns and fragment ion ratio at collision energy of -27 eV.

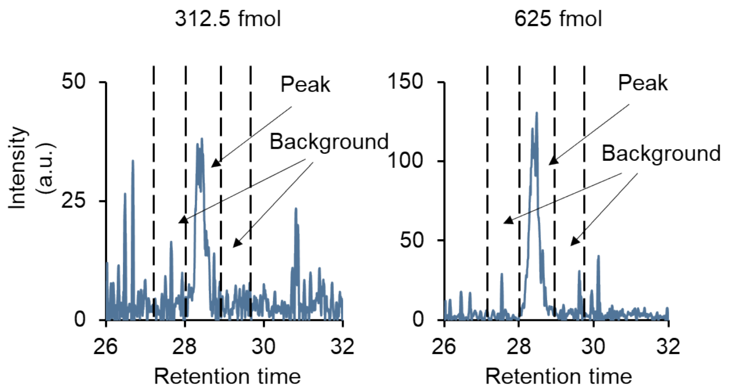

Figure S2. Limit of detection and limit of quantification of PtdIns(3,4,5)P_3. The XIC of fragment ion of GroPIns(3,4,5)P_3 at 321 m/z where 3.125 pmol and 6.25 pmol PtdIns(3,4,5)P_3 standard were spiked into unstimulated human platelets extract, with 1/10 of the sample injected into the column. Signal to noise ratio = 3.9 for 312.5 fmol and 12.5 for 625 fmol.

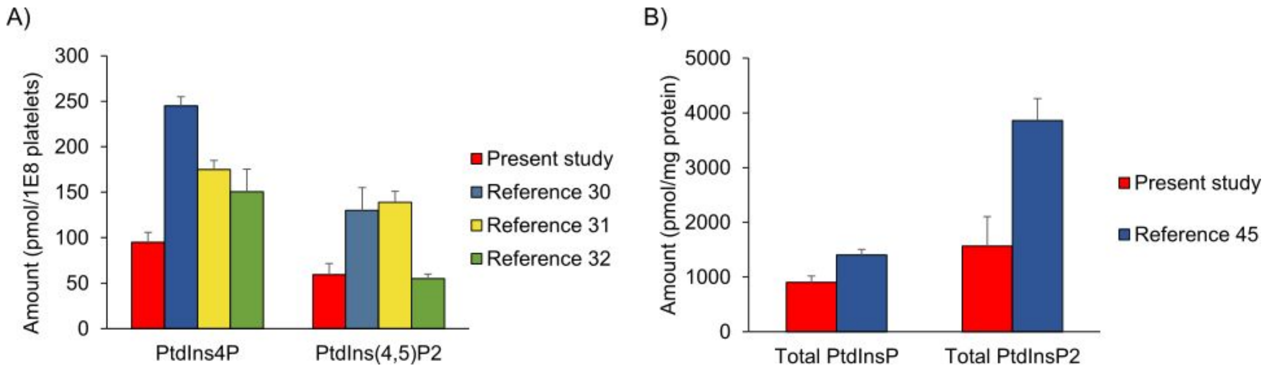

Figure S3. Comparison of amount of the identified phosphoinositides species with literature. The phosphoinositides quantified in (A) unstimulated human platelets and (B) hippocampus heavy membrane fraction were compared with previous reports. The results showed that the findings in the present study were consistent with previous studies.

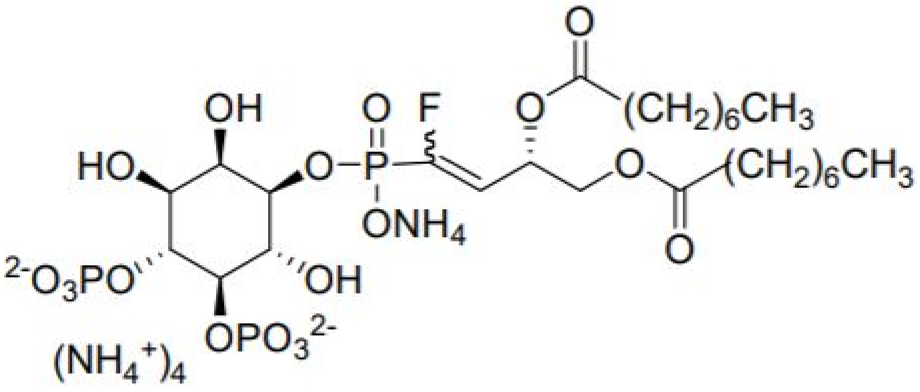

Figure S4. Structure of internal standard 16:0/16:0 PtdIns(4,5)P2 α-fluorovinyl-phosphonate (PtdIns(4,5)P2-FP).

Species	Q1 Mass (m/z)	Q3 Mass (m/z)	Collision energy (eV)	Monitoring window (min)
Glycerophosphoinositol phosphate (GroPInsP)	413	241	-27	11 - 17
Glycerophosphoinositol bisphosphate ($GroPInsP_2$)	493	395	-27	21 - 25
Glycerophosphoinositol triphosphate ($GroPInsP_3$)	573	321	-32	26 - 32
Glycerophosphoinositol (4,5)-bisphosphate α-fluorovinyl-phosphonate ($GroPIns(4,5)P_2$-FP)	509	411	-27	21 - 25

Table S1. SRM transition list for the current study. SRM transition list used for glycerophosphoinositol phosphate analysis.
